# A rare suprasellar lesion with multifocal central nervous system involvement in clinically diagnosed von Hippel-Lindau disease: A case report

**DOI:** 10.3892/etm.2026.13294

**Published:** 2026-09-11

**Authors:** Zekai Wu, Lei Zhang, Meng Tan, Laian Zhang

**Affiliations:** Department of Radiology, Jiangyou People's Hospital, Jiangyou, Sichuan 621700, P.R. China

**Keywords:** von Hippel-Lindau disease, hemangioblastoma, suprasellar lesion, MRI, case report

## Abstract

Von Hippel-Lindau (VHL) disease is a rare autosomal dominant disorder characterized by a predisposition to multiple tumors, including central nervous system (CNS) hemangioblastomas, retinal hemangioblastomas and renal cell carcinoma. The present report details the case of a 39-year-old male patient presenting with progressive dizziness and left-sided visual deterioration, whose neuroimaging results revealed multifocal hypervascular lesions in the suprasellar region, left cerebellar hemisphere and spinal cord. Following resection of the cerebellar lesion, histopathological examination confirmed the diagnosis of hemangioblastoma, and the overall pattern of multifocal CNS involvement supported a clinical diagnosis of VHL disease. This case highlighted a rare suprasellar manifestation of VHL disease and the diagnostic challenges posed by atypical multifocal CNS presentations.

## Introduction

Von Hippel-Lindau (VHL) disease (Online Mendelian Inheritance in Man identifier, #193300) is a rare autosomal dominant tumor-predisposition syndrome that is caused by pathogenic germline variants of the VHL tumor suppressor gene, which is located on chromosome 3p25-26. VHL exhibits an estimated prevalence of 1 in 39,000-91,000 individuals and a birth incidence of 1 in 36,000-45,500 live births (1). Clinical conditions associated with VHL disease include retinal and central nervous system (CNS) hemangioblastomas, clear cell renal cell carcinoma, pheochromocytoma, pancreatic neuroendocrine and endolymphatic sac tumors, and epididymal and broad ligament cystadenomas (2). CNS hemangioblastomas most commonly arise in the cerebellum and spinal cord (3). In particular, suprasellar hemangioblastomas are CNS hemangioblastomas that remain notably rare and may represent an uncommon manifestation of VHL disease (4).

For example, a study by Filizoglu and Ozguven (5) reported a histologically confirmed suprasellar hemangioblastoma in a 52-year-old male patient with established VHL disease. The lesion demonstrated notable gallium-68 DOTA-Tyr^3^-octreotate uptake, highlighting the potential role of somatostatin receptor PET/CT in the management of VHL disease. The present case differed from the aforementioned report by demonstrating concurrent involvement of the suprasellar region, cerebellum and spinal cord in a patient with hemangioblastoma who did not have a known family history or molecular confirmation of VHL disease. The cerebellar lesion observed in the present patient was histopathologically confirmed to be a hemangioblastoma, but the suprasellar lesion was radiologically presumed as a hemangioblastoma; direct sampling of the suprasellar lesion was not pursued due to its marked vascularity, encasement of the left internal carotid artery and displacement of the optic chiasm. The present case illustrated how integrated MRI findings across multiple CNS compartments, together with pathological confirmation of the tumor type from a surgically accessible lesion, can support a clinical diagnosis of VHL disease and guide clinical management strategies in cases where biopsy or resection of a highly vascular suprasellar lesion carries notable vascular and visual risks.

## Case report

A 39-year-old male patient was admitted to the Department of Neurosurgery of Jiangyou People's Hospital (Jiangyou, China) in November 2024, with a 1-month history of positional dizziness that was aggravated by standing and relieved by rest, which was also accompanied by progressive painless visual blurring in the left eye. The patient denied experiencing headaches, nausea, vomiting, gait imbalance, limb weakness, sensory disturbance or seizures. The patient had undergone resection of a cerebellar lesion at another institution 14 years earlier, but detailed clinical records and histopathological findings for this prior lesion were unavailable. The patient had no history of hypertension, diabetes mellitus, cardiovascular disease, chronic infection or drug allergy. However, the patient did have a 15-pack-year smoking history and reported occasional alcohol consumption. The family history of the patient was negative for hereditary disorders, including VHL disease, across three generations.

On admission, the patient exhibited tachycardia (111 bpm) and a blood pressure of 114/90 mmHg. The results of general physical examinations, including assessment of the patient's general condition and cardiovascular, respiratory and abdominal systems, as well as other routine systemic examinations, were otherwise unremarkable. The results of neurological examinations, including assessment of consciousness, pupillary responses, visual function, motor and sensory function, deep tendon reflexes and coordination and balance, showed that the patient was alert and oriented, and the patient scored a Glasgow Coma Scale score of 15(6). The patient exhibited equally sized pupils at 2.5 mm bilaterally and normal pupillary reflexes were observed. The patient also exhibited a corrected visual acuity of 20/40 in the left eye, and confrontation visual fields showed no visual field defects. The results of motor examinations, including assessment of muscle strength, muscle tone and muscle bulk, showed that the patient exhibited full strength in all muscle groups, exhibiting grade 5 on the Medical Research Council Muscle Scale (7), with normal muscle tone and bulk. Sensory examination included assessment of superficial sensation (light touch, pinprick and temperature) and deep sensation (vibration and joint position sense), with no abnormalities identified bilaterally. Deep tendon reflexes were symmetrical and graded as 2+ (normal) (8), and no pathological reflexes were elicited. Cerebellar examinations revealed a positive Romberg test result and mild bilateral dysmetria during finger-to-nose testing. Notably, meningeal signs were absent and ophthalmologic examinations after pupillary dilatation revealed no evidence of retinal hemangioblastomas. Abdominal CT and ultrasonography revealed a polypoid lesion in the gallbladder, with no renal, adrenal or pancreatic abnormalities suggestive of VHL disease. Scrotal and epididymal ultrasonography revealed no abnormalities suggestive of VHL disease. Routine laboratory examinations including liver and renal function tests, specifically alanine aminotransferase, aspartate aminotransferase, total bilirubin, serum creatinine and blood urea nitrogen, showed no clinically relevant abnormalities.

MRI was performed before resection of the cerebellar lesion using a 1.5-T scanner, with contrast-enhanced images obtained after intravenous administration of 0.1 mmol/kg gadopentetate dimeglumine. MRI scans revealed multiple lesions in the suprasellar region, left cerebellar hemisphere and spinal cord. In the left cerebellar hemisphere, a large cystic lesion with smooth walls measuring 5.9x4.6x3.5 cm was observed, showing hypointensity in T1-weighted images and hyperintensity in T2-weighted scans (Fig. 1A and B). The lesion contained a posterolateral mural nodule that exhibited mild hypointensity in T1-weighted images and hyperintensity in T2-weighted images (Fig. 1A and B), with no diffusion restriction observed on diffusion-weighted imaging (Fig. 1D). Sagittal T2-weighted imaging further demonstrated the cystic lesion with its mural nodule (Fig. 1F). Following contrast administration, the mural nodule showed a marked increase in signal intensity on post-contrast T1-weighted images, indicating marked contrast enhancement consistent with the hypervascular nature of the lesion (Fig. 1E), with notable perilesional edema on T2-weighted fluid-attenuated inversion recovery (FLAIR) images (Fig. 1C) causing compression of the dorsolateral brainstem and effacement of the fourth ventricle. In the suprasellar region, a solid nodule measuring 2.3x2.0x2.3 cm was identified, which showed heterogeneous hypointensity in T1-weighted images (Fig. 2A) and hyperintensity in T2-weighted and T2-weighted FLAIR images (Fig. 2B and C). No diffusion restriction was observed on diffusion-weighted imaging. After contrast administration, the suprasellar lesion showed notable homogeneous signal enhancement (Fig. 2D-F). This lesion was also shown to contain multiple serpentine flow voids (Fig. 2B and C), which were more conspicuous on contrast-enhanced images compared with initial MRI scans (Fig. 2D-F). The suprasellar lesion encased the left internal carotid artery and displaced the optic chiasm. Spinal cord imaging revealed intramedullary signal abnormalities extending from the T3 to T9 vertebral levels, which appeared hypointense in T1-weighted images (Fig. 3A) and hyperintense in T2-weighted and short TI inversion recovery images (Fig. 3B and C). The background spinal cord-signal abnormalities did not show marked enhancement following contrast administration (Fig. 3D); however, within these abnormalities, discrete nodules showing marked contrast enhancement were identified at the T5 and T7 vertebral levels (Fig. 3E and F), but these lesions were not subjected to biopsy or histopathological examination. Despite the aforementioned spinal MRI findings, no clinical symptoms or neurological signs specifically attributable to the T5 and T7 lesions were identified in the patient; lower-extremity strength and sensation were preserved, deep tendon reflexes were symmetrical, and no pathological reflexes were observed. The overall imaging pattern was suggestive of VHL disease, given the multifocal CNS lesions involving the cerebellum, suprasellar region and spinal cord with imaging features suggestive of hemangioblastomas.

Given the observations of notable brainstem compression, microsurgical resection of the left cerebellar lesion was prioritized and performed 4 days after the patient's admission. By contrast, the characteristics of the suprasellar lesion, which displayed notable encasement of the left internal carotid artery, marked vascularity and a close relationship with the displaced optic chiasm, markedly increased the risks of vascular injury, intraoperative hemorrhage and visual pathway damage occurring during biopsy. Accordingly, biopsy or resection of this lesion was not pursued, and MRI surveillance was recommended. Intraoperatively, cerebellar tissue herniation was observed ~1.5 cm above the bone edge and was resolved following aspiration of 20 ml light yellow cystic fluid. The decompression achieved by aspiration restored parenchymal pulsation and exposed the mural nodule of the aforementioned left cerebellar lesion, which was a well-demarcated hypervascular tumor measuring 1.6 cm in diameter. The tumor was located adjacent to the transverse and sigmoid sinuses and caused marked compression of the surrounding neural structures. Gross total resection was achieved under microscopic visualization using a light microscope, followed by hemostasis and placement of a synthetic dural graft. As shown in Fig. 4, the resected specimen was a well-circumscribed reddish solid tumor nodule.

As demonstrated in Fig. 5, histopathological examinations of the resected lesion confirmed the diagnosis of grade 1 hemangioblastoma, in accordance with the 2021 Classification of Central Nervous System Tumors guidelines published by the World Health Organization (9). Following resection, the specimen was immediately sent to the pathology department and fixed in 10% neutral buffered formalin at room temperature for 24 h, routinely paraffin-embedded and sectioned at a thickness of 3-4 µm. H&E staining was performed at room temperature, with sections stained with hematoxylin for 5 min and eosin for 1 min, following the aforementioned fixation in 10% neutral buffered formalin and routine paraffin processing. For immunohistochemistry, heat-induced antigen retrieval was performed using EDTA antigen retrieval working solution (pH 9.0) at 100˚C prior to antibody staining. Prior to primary antibody incubation, endogenous peroxidase activity was blocked using 3% hydrogen peroxide at room temperature for 5 min. No separate non-specific protein blocking step was performed prior to primary antibody incubation. The following ready-to-use primary antibodies were used: CD34 (clone QBEnd/10; cat. no. Kit-0004; Fuzhou Maixin Biotechnology Development Co., Ltd.), S-100 (clone SD16; cat. no. AR0210; Xiamen Talent Biomedical Technology Co., Ltd.), Ki-67 (clone BP6045; cat. no. I10092E-01; Tuling Biomedical Co., Ltd.) and vimentin (clone BP6010; cat. no. I10032E-01; Tuling Biomedical Co., Ltd.). A ready-to-use secondary antibody immunohistochemical detection reagent (cat. no. DD23; Xiamen Talent Biomedical Technology Co., Ltd.) and DAB chromogen supplied by Xiamen Talent Biomedical Technology Co., Ltd. were used. Immunohistochemical staining was performed using a TALENT 144 fully automated immunohistochemistry stainer (Xiamen Talent Biomedical Technology Co., Ltd.) according to the manufacturer's preset protocol. The stained sections were examined using a Nikon Ci-S light microscope (Nikon Corporation). H&E staining demonstrated abundant delicate capillaries embedded in a fine fibrillary stroma (Fig. 5A), with intervening stromal cells showing foamy cytoplasm and vacuolated nuclei (Fig. 5B). Immunohistochemical analysis revealed the notable expression of CD34 in endothelial cells (Fig. 5C) and the expression of S-100 and vimentin in stromal cells (Fig. 5D and E). The Ki-67 labeling index was determined by manual counting of positively stained tumor cells under light microscopy and was ~5% (Fig. 5F). Overall, the histomorphological and immunophenotypical findings of the present case were compatible with a hemangioblastoma diagnosis. Immunostaining for inhibin-α was not performed as part of the diagnostic panel, although it may serve as an ancillary marker for hemangioblastoma (10).

Although genetic counseling and VHL gene mutation analysis were recommended to the patient in order to confirm the diagnosis, guide long-term surveillance strategies and facilitate family screening for VHL, the patient declined genetic testing despite counseling regarding its potential clinical and familial benefits. Genetic counseling for potentially at-risk family members remained advisable, as early detection of VHL-associated tumors has been shown to improve clinical outcomes (11). Postoperatively, the patient recovered satisfactorily, with notable improvements in dizziness, and was discharged on postoperative day 7 with recommendations for regular surveillance imaging and multidisciplinary follow-up. No additional adjuvant treatment was administered following resection of the cerebellar lesion, while the suprasellar and spinal lesions were managed conservatively, with imaging surveillance recommended. At the 3-month multidisciplinary follow-up appointment, the patient remained clinically stable, with no new or worsening neurological symptoms, but declined the recommended surveillance imaging and expressed reluctance to participate in long-term surveillance. Therefore, the subsequent radiographic status of the suprasellar and spinal lesions could not be assessed.

## Discussion

Clinical diagnosis of VHL disease are based on any of the following three criteria: i) A positive family history plus at least one characteristic VHL-associated tumor, such as a retinal or CNS hemangioblastoma, clear cell renal cell carcinoma, pancreatic neuroendocrine tumor or endolymphatic sac tumor; ii) the presence of two or more retinal or CNS hemangioblastomas; or iii) one retinal or CNS hemangioblastoma together with at least one characteristic visceral VHL-associated tumor, excluding renal and epididymal cysts (12).

In the present case, the patient exhibited no known family history of VHL and was diagnosed with histologically confirmed cerebellar hemangioblastoma, whereas the suprasellar lesion observed in the present case was radiologically presumed. The combination of lesions observed in the present case, including a histologically confirmed cerebellar hemangioblastoma, a radiologically presumed suprasellar hemangioblastoma and spinal-cord lesions with imaging features characteristic of hemangioblastoma, represented a rare manifestation of VHL disease. The imaging findings for spinal lesions were markedly suggestive of hemangioblastoma, including discrete contrast-enhancing intramedullary nodules within non-enhancing T2-hyperintense spinal cord-signal abnormalities (13). Although the suprasellar and spinal lesions were not histologically confirmed, their characteristic vascular imaging patterns, together with the presence of pathology-confirmed cerebellar hemangioblastoma, supported the clinical diagnosis of multifocal VHL disease (14). The absence of genetic confirmation for VHL disease remains a limitation of the present case, as it precluded definitive molecular diagnosis and cascade screening for at-risk family members. According to the established clinical diagnostic criteria (15), the combination of a histologically confirmed cerebellar hemangioblastoma and additional CNS lesions with characteristic VHL-associated imaging features supported a clinical diagnosis of VHL disease, even in the absence of genetic testing.

Hemangioblastomas are vascular CNS tumors that typically appear as well-defined masses in conventional imaging results (16). These tumors are generally classified into three types based on imaging features: Simple cysts, large cysts with small mural nodules and solid or predominantly solid nodules with minimal cystic change (17). Although hemangioblastomas may occur throughout the CNS, they predominantly arise in the cerebellum, brainstem and spinal cord (17). Suprasellar involvement is rare. Given the notable surgical risk associated with the suprasellar lesion, biopsy or resection of this lesion was not pursued in the present case.

Notably, in the present case, the suprasellar lesion demonstrated characteristic imaging features of hemangioblastoma, particularly marked hypervascularity, notable homogeneous enhancement of the lesion following contrast administration compared with pre-contrast images and serpentine flow voids. The predominant differential diagnoses for a hypervascular suprasellar lesion include: i) Pituitary macroadenoma; ii) angiomatous meningioma; and iii) papillary craniopharyngioma (18). Pituitary macroadenomas, the most common sellar tumors, typically extend superiorly through the sellar diaphragm, producing a characteristic ‘snowman’ configuration. In MRI scans, pituitary macroadenomas usually appear isointense relative to gray matter in T1-weighted images and demonstrate variable signal intensity in T2-weighted imaging results. Following contrast administration, the solid components of pituitary macroadenomas typically exhibit a less intense and often heterogeneous increase in signal intensity than the normal pituitary gland, although cystic degeneration and hemorrhage may be present (19,20). However, pituitary macroadenomas do not typically demonstrate the notable serpentine flow voids or marked hypervascular features observed in hemangioblastomas. Although rare hypervascular adenomas may exhibit flow voids, the combination of notable serpentine flow voids, marked contrast enhancement and the absence of typical sellar expansion or obvious dural attachment and bone involvement favored a radiological diagnosis of suprasellar hemangioblastoma in the present case (21).

Angiomatous meningiomas are typically dural-based tumors that show notable contrast enhancement and MRI scans of these tumors may contain intratumoral or peritumoral flow voids. However, these tumors usually exhibit broad dural attachment, which is often accompanied by dural thickening, as evidenced by the presence of the dural tail sign in MRI scans, hyperostosis or adjacent bone remodeling, features that were absent in the present case (18). Papillary craniopharyngiomas usually develop in adults and are typically solid lesions showing contrast enhancement without calcification, although calcification is commonly observed in tumors of the adamantinomatous subtype (22). However, the marked hypervascularity and serpentine flow voids observed in the present case are atypical features of craniopharyngiomas, making this diagnosis improbable. As such, the combination of marked hypervascularity, serpentine flow voids and the absence of obvious dural attachment or bone involvement in the suprasellar lesion in the present case favored a radiological diagnosis of suprasellar hemangioblastoma. Together with the presence of a histologically confirmed cerebellar hemangioblastoma and the characteristic spinal-cord imaging findings of hemangioblastomas observed in the present case, these features supported the clinical diagnosis of VHL disease.

The current management of VHL-associated hemangioblastomas primarily involves surgical resection. This aims to achieve gross total tumor removal with minimal permanent postoperative neurological deficits, particularly for cerebellar and spinal lesions (23). Concurrently, resection of VHL-associated hemangioblastomas aims to prevent acute obstructive hydrocephalus, which is a major cause of central nervous system mortality that occurs in ~28% of symptomatic cases (24). Preoperative embolization may reduce the risk of intraoperative bleeding in highly vascular lesions, but the potential benefits of this strategy must be weighed against procedure-related risks (25). Stereotactic radiosurgery represents an alternative approach for inoperable multifocal or recurrent tumors, although assessing the efficacy of this strategy remains challenging because of natural growth-arrest patterns, which can make it difficult to distinguish treatment-related tumor control from spontaneous growth stabilization (26).

In the present case, comprehensive abdominal evaluations revealed no renal, adrenal, pancreatic or genital manifestations suggestive of VHL. However, lifelong multidisciplinary abdominal-tumor surveillance remains important in patients with VHL disease; this is because clear cell renal cell carcinoma, for which the lifetime risk of ~70% in patients with VHL disease, represents the leading cause of VHL-related mortality, and untreated pheochromocytoma crises and metastatic pancreatic neuroendocrine tumors also contribute notably to mortality in patients with VHL disease (27,28). As such, abdominal-tumor surveillance should include abdominal regular MRI and annual catecholamine screening to reduce non-neurological mortality (29-31).

The present patient displayed a histologically confirmed cerebellar hemangioblastoma, spinal cord lesions with characteristic MRI features of hemangioblastomas and a solid suprasellar nodule exhibiting notable homogeneous enhancement following contrast administration and serpentine flow voids. Taken together, these findings strongly supported a clinical diagnosis of VHL disease. Although histological verification of all lesions would have been ideal, the convergence of pathology-proven cerebellar hemangioblastoma and characteristic hemangioblastoma-associated neuroimaging findings provided notable diagnostic evidence indicative of VHL disease. Genetic testing would have provided a definitive molecular diagnosis and enabled the cascade screening of family members; however, the patient declined such testing, thereby limiting the completeness of the diagnostic workup and precluding family-level genetic counseling. Given the high mortality risk associated with renal cell carcinoma and pheochromocytoma, in addition to those associated with CNS manifestations, lifelong surveillance for these conditions remains important (32). Despite counseling regarding the risks of recurrence, progression and the development of additional VHL-associated tumors, the patient remained reluctant to undergo long-term surveillance, which limited the assessment of subsequent disease evolution.

In conclusion, the present case highlighted a rare suprasellar lesion in clinically diagnosed VHL disease with multifocal central nervous system involvement. Together with the histologically confirmed cerebellar hemangioblastoma and spinal-cord imaging findings characteristic of hemangioblastomas, the suprasellar lesion supported a clinical diagnosis of VHL disease and broadened the differential diagnosis of hypervascular sellar and parasellar masses in patients with VHL disease. In future cases where VHL disease is clinically suspected, hemangioblastoma should be considered in the differential diagnosis of atypical sellar or parasellar lesions.

## Figures and Tables

**Figure 1:**
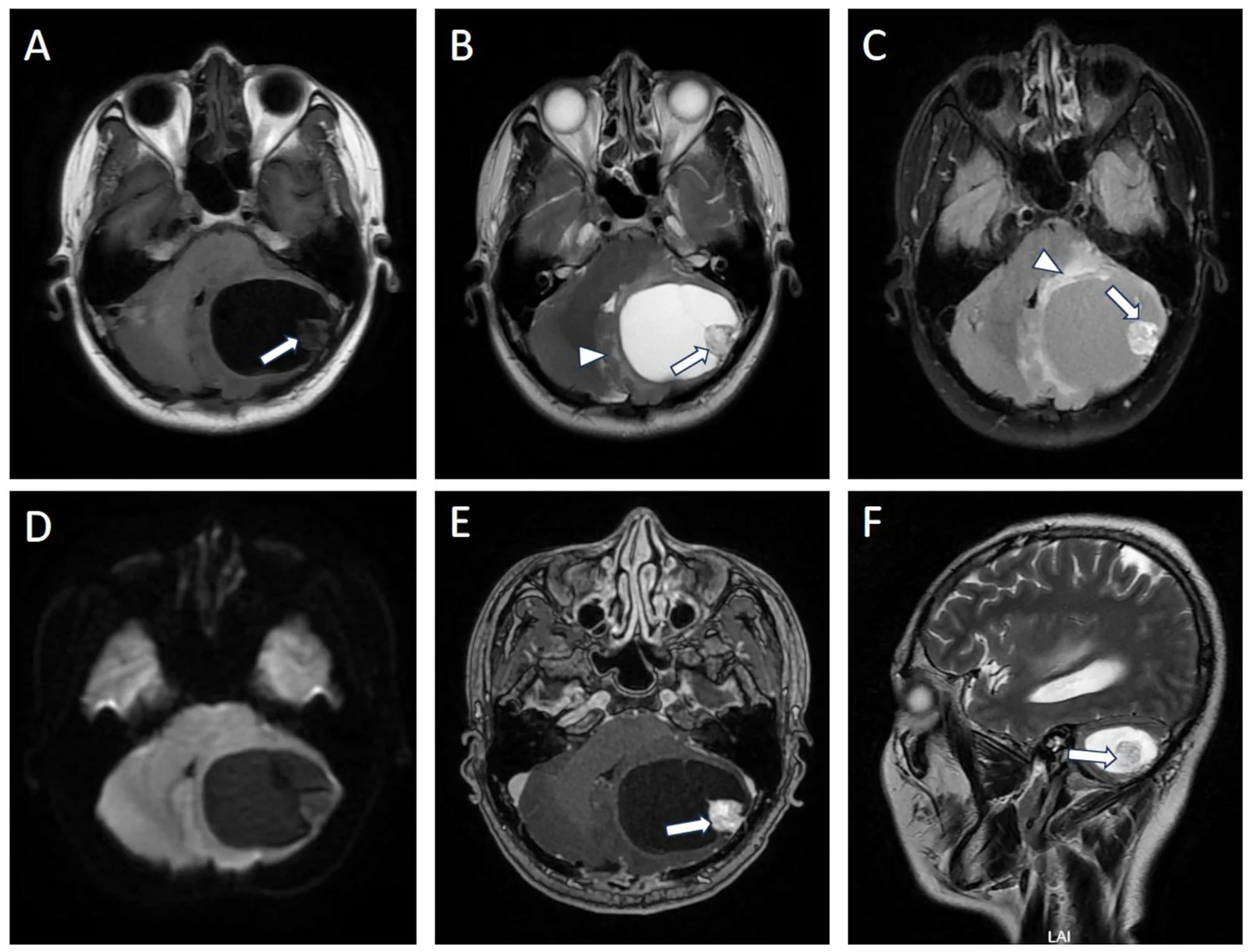
Preoperative MRI scans of the left cerebellar hemangioblastoma. (A) Axial T1-weighted image showing a hypointense cystic lesion in the left cerebellum. (B) Axial T2-weighted image demonstrating cystic hyperintensity in the cerebellar lesion with a posterolateral mural nodule. (C) T2-weighted fluid-attenuated inversion recovery image revealing perilesional edema. (D) Diffusion-weighted imaging indicated a lack of diffusion restriction. (E) Axial contrast-enhanced T1-weighted image showing marked enhancement of the mural nodule. (F) Sagittal T2-weighted image demonstrating the cystic lesion with a mural nodule. The lesion caused compression of the dorsolateral brainstem and effacement of the fourth ventricle. Arrows indicate the mural nodule, and arrowheads indicate perilesional edema.

**Figure 2:**
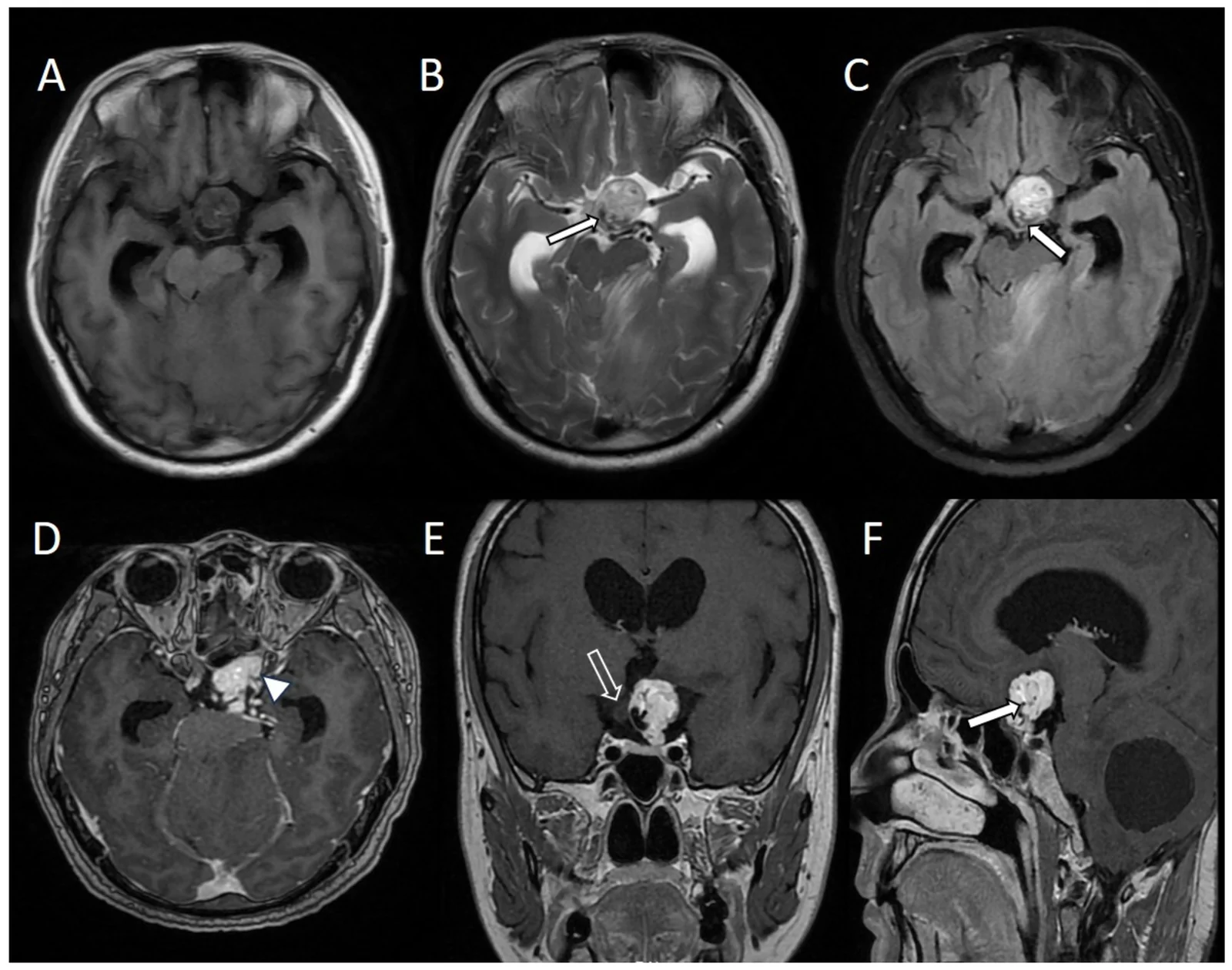
Preoperative MRI scans of the suprasellar lesion. (A) Axial T1-weighted image depicting a heterogeneously hypointense suprasellar mass. (B) Axial T2-weighted image showing hyperintense signals for this lesion. (C) T2-weighted fluid-attenuated inversion recovery image showing that the lesion exhibited intrinsic hyperintensity. Contrast-enhanced (D) axial, (E) coronal and (F) sagittal images demonstrating that the lesion exhibited homogeneous signal enhancement, encasement of the left internal carotid artery and displacement of the optic chiasm. Filled arrows indicate flow voids, the arrowhead indicates encasement of the left internal carotid artery and the empty arrow indicates displacement of the optic chiasm.

**Figure 3:**
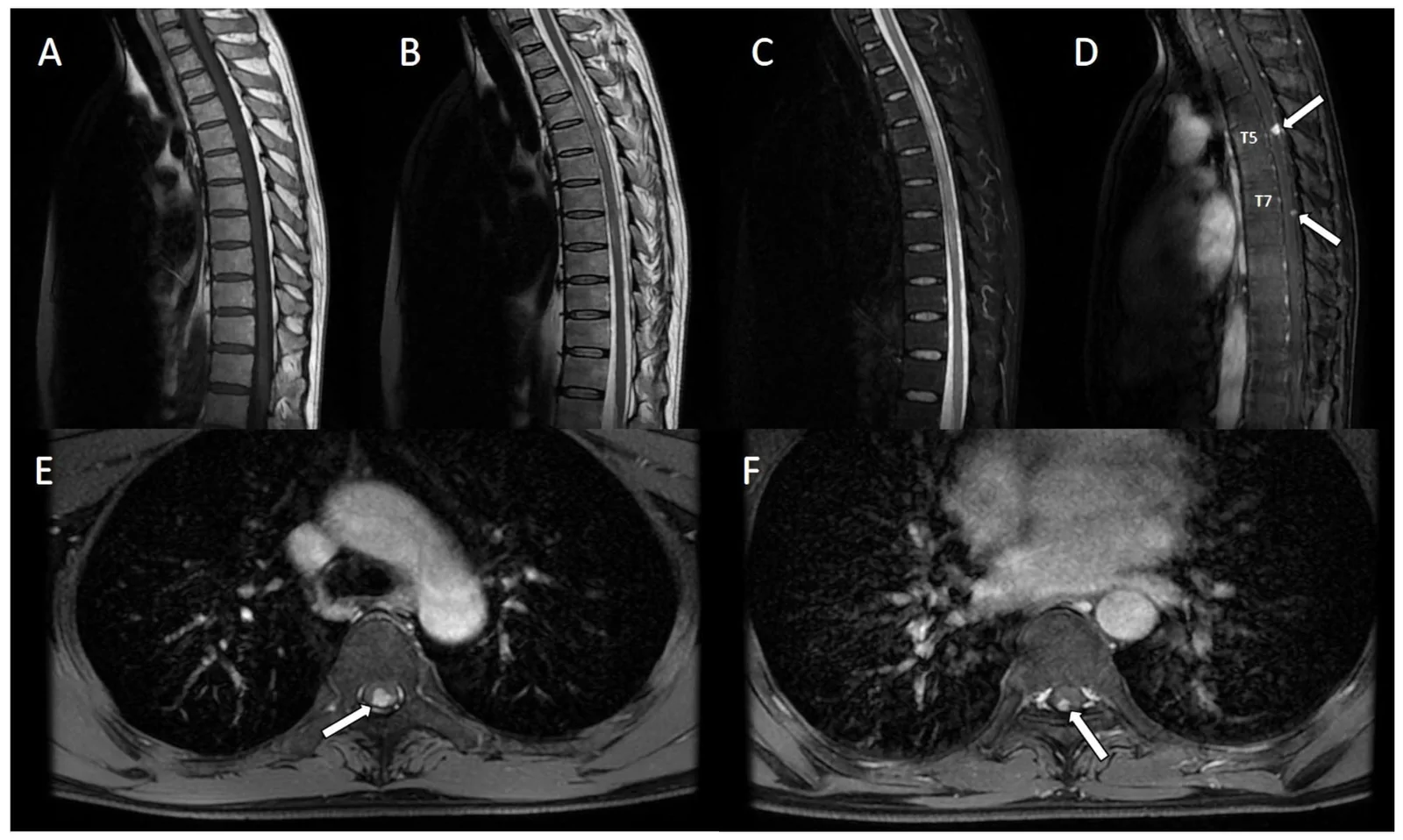
Spinal MRI scans of the intramedullary lesions. (A) Sagittal T1-weighted image revealing a strip-like slightly hypointense signal extending from the T3 to T9 vertebral levels. The corresponding slightly hyperintense signal is observed in (B) T2-weighted and (C) short TI inversion recovery images. (D) Sagittal T1-weighted contrast-enhanced image demonstrating contrast enhancement of the intramedullary nodules, as indicated by arrows within the figure. Axial T1-weighted contrast-enhanced images at the (E) T5 and (F) T7 vertebral levels confirmed the presence of well-defined, contrast-enhancing intramedullary nodules, which are indicated by arrows within the figure.

**Figure 4:**
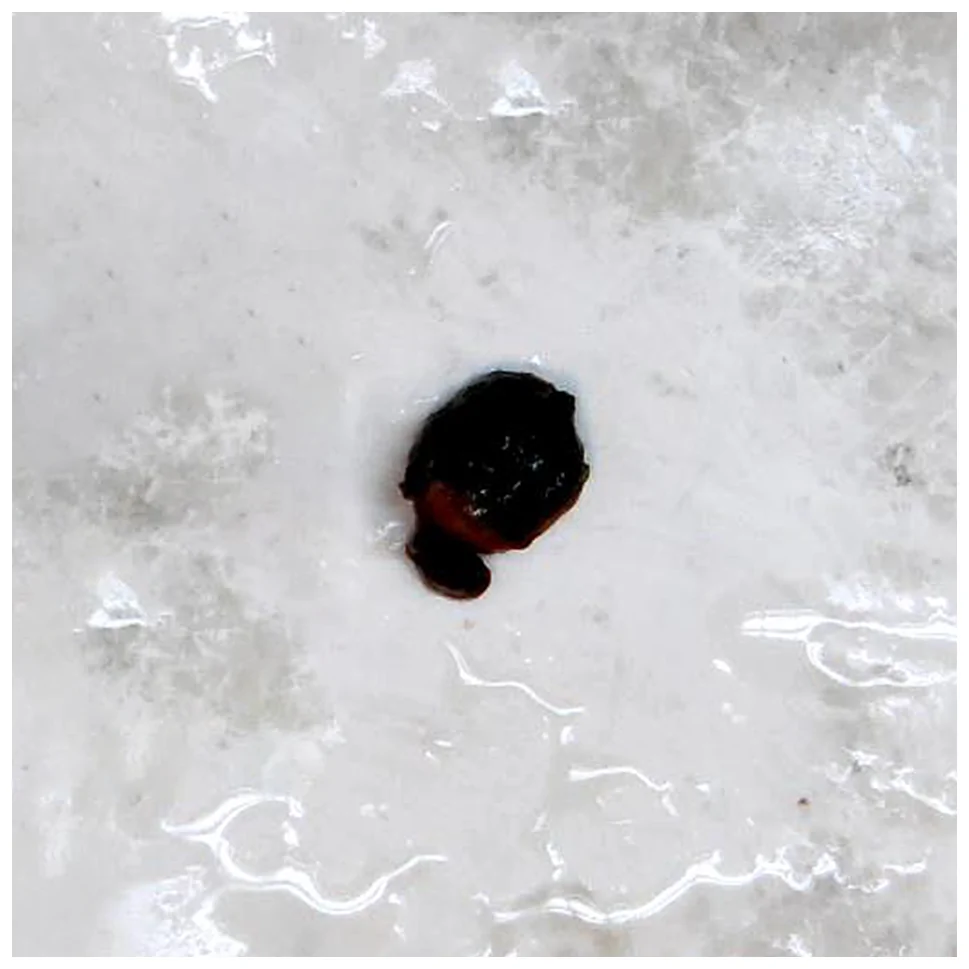
Representative image of the resected cerebellar hemangioblastoma. The well-circumscribed, solid tumor nodule, measuring 1.6 cm in diameter, shows a characteristic reddish-tan cut surface with a rich vascular appearance.

**Figure 5:**
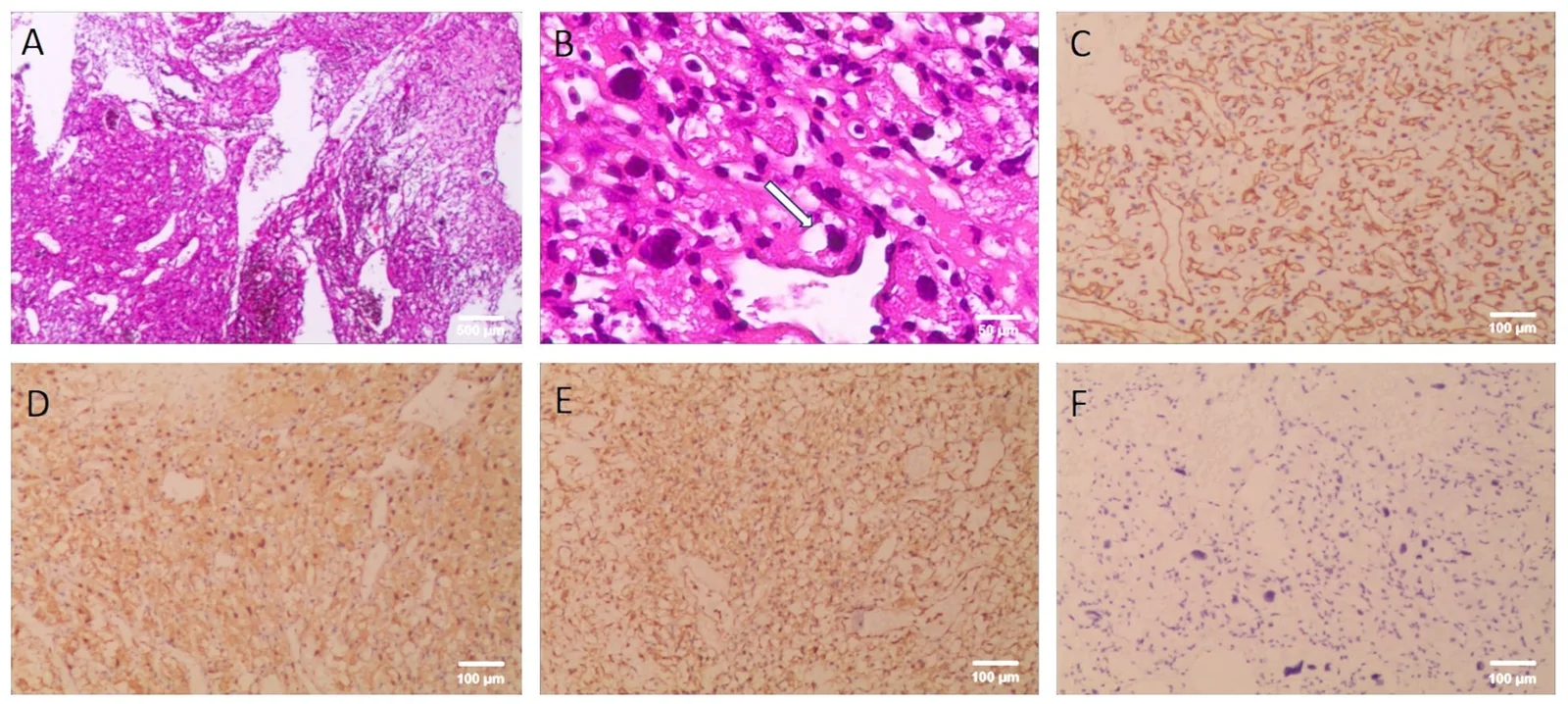
Histopathological and immunohistochemical features of the resected cerebellar hemangioblastoma. (A) H&E staining (x40 magnification; scale bar, 500 µm) revealed abundant capillaries embedded within a fine fibrillary stroma. (B) H&E staining (x400 magnification; scale bar, 50 µm) showed stromal cells with foamy cytoplasm and vacuolated nuclei. Immunohistochemistry (x200 magnification; scale bar, 100 µm) revealed (C) CD34 positivity in endothelial cells and (D) S-100 and (E) vimentin positivity in stromal cells. (F) Immunohistochemical staining for Ki-67 (x200 magnification; scale bar, 100 µm) also revealed a Ki-67 labeling index value of ~5%. Arrows indicate vacuolated nuclei.

## Data Availability

The data generated in the present study may be requested from the corresponding author.
